# Supplementary material for: Characterizing uncertainty in predictions of genomic sequence-to-activity models
Source: bioRxiv. 2023 Dec 23:2023.12.21.572730. Preprint. [Version 1] doi: 10.1101/2023.12.21.572730 (PMC10769392; doi:10.1101/2023.12.21.572730)
Supplement: Supplement 1 [file NIHPP2023.12.21.572730v1-supplement-1.pdf]

bioRxiv preprint doi: https://doi.org/10.1101/2023.12.21.572730; this version posted December 23, 2023. The copyright holder for this preprint (which was not certified by peer review) is the author/funder, who has granted bioRxiv a license to display the preprint in perpetuity. It is made available under aCC-BY 4.0 International license.

A Appendix

Supplementary Figures

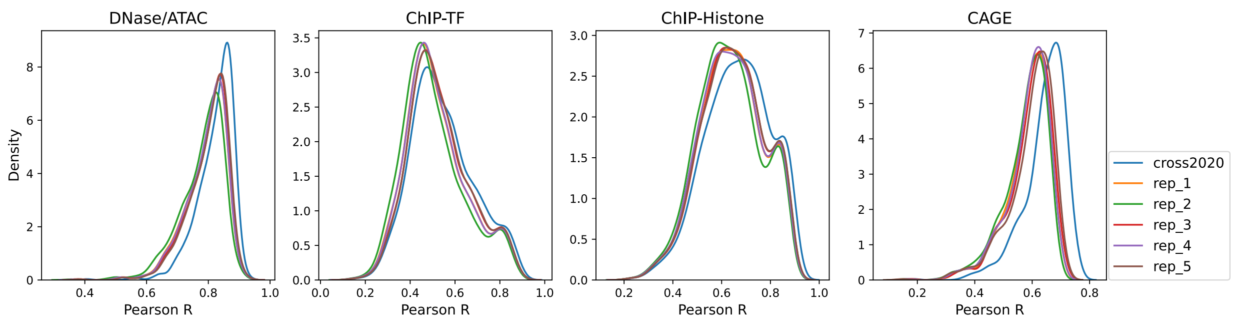

Figure S1: **Test set performance of replicates.** Pearson correlation on held-out test data across prediction tracks for each assay.

bioRxiv preprint doi: https://doi.org/10.1101/2023.12.21.572730; this version posted December 23, 2023. The copyright holder for this preprint (which was not certified by peer review) is the author/funder, who has granted bioRxiv a license to display the preprint in perpetuity. It is made available under aCC-BY 4.0 International license.

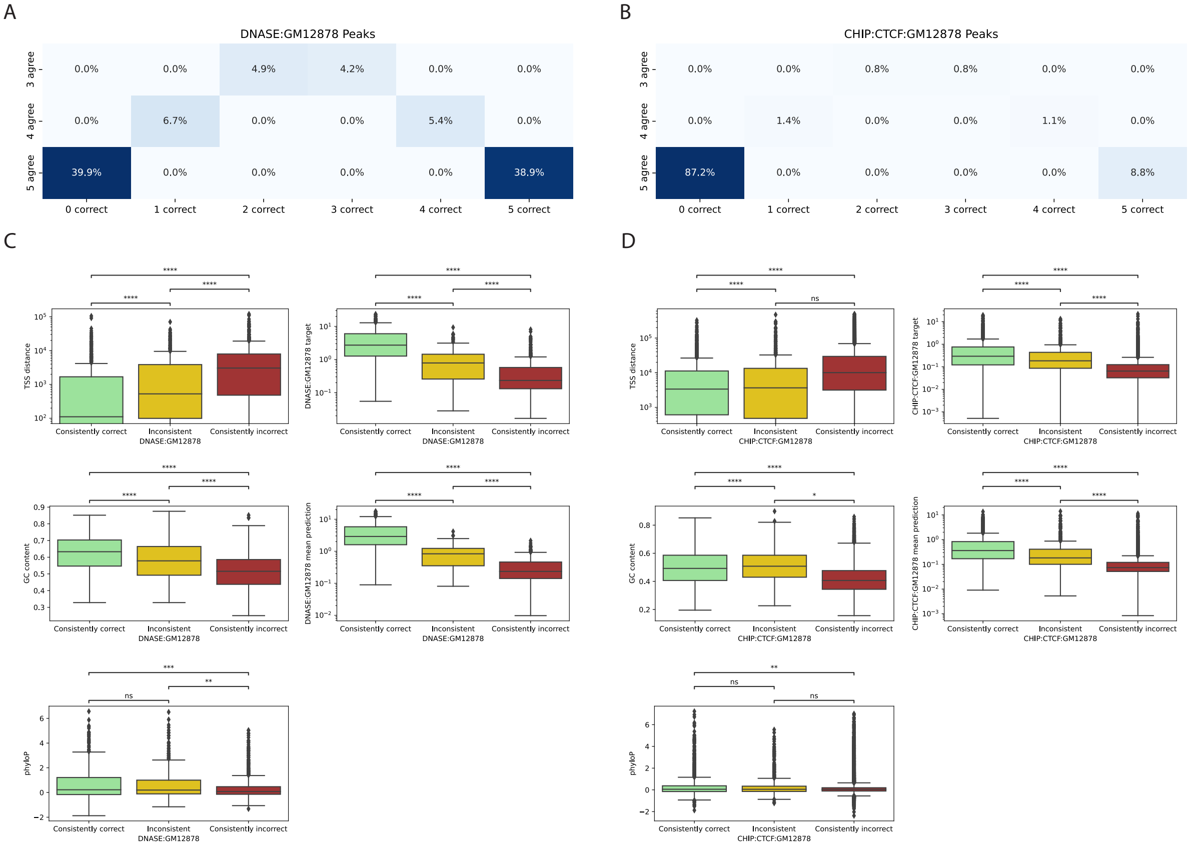

Figure S2: **Consistently correctly predicted peaks are gene-proximal, have higher GC content, and are evolutionarily conserved.** For (a) DNase-seq and (b) CTCF ChIP-seq in GM12878 cells, we display heatmaps of prediction consistency and correctness across peak sequences. For (c) DNase-seq and (d) CTCF ChIP-seq in GM12878 cells, we measure differences in the sequences falling into each of the three consistency categories (consistently correct vs. inconsistent vs. consistently incorrect) across five attributes – TSS distance, GC content, evolutationary conservation (phyloP), experimentally measured activity level (target) and mean predicted activity level.

bioRxiv preprint doi: https://doi.org/10.1101/2023.12.21.572730; this version posted December 23, 2023. The copyright holder for this preprint (which was not certified by peer review) is the author/funder, who has granted bioRxiv a license to display the preprint in perpetuity. It is made available under aCC-BY 4.0 International license.

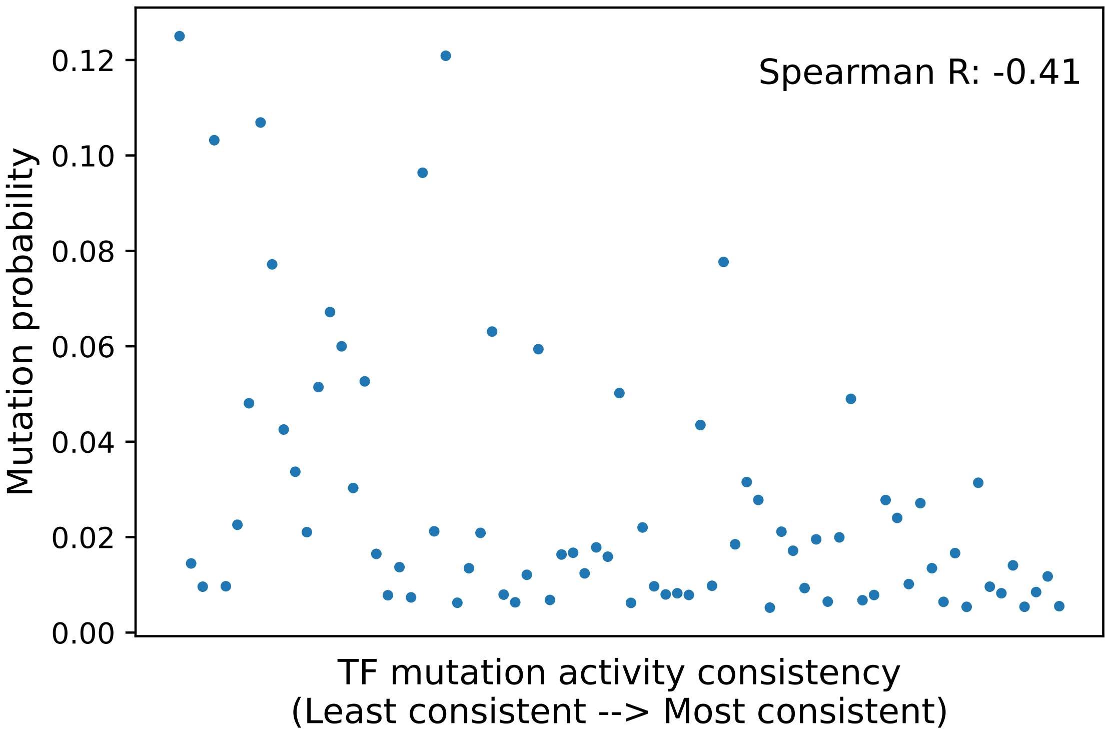

Figure S3: **Higher probability (less disruptive) mutations to TF motifs have less consistent predictions across replicates.** For tested mutations to TF motifs with probability greater than 0.005 according to the TF's PWM, we plot consistency in the TF mutation activity scores (calculated using perturbations 10bp upstream of the TSS) across replicates versus mutation probability. We observe that higher probability mutations have less consistent predictions.

bioRxiv preprint doi: https://doi.org/10.1101/2023.12.21.572730; this version posted December 23, 2023. The copyright holder for this preprint (which was not certified by peer review) is the author/funder, who has granted bioRxiv a license to display the preprint in perpetuity. It is made available under aCC-BY 4.0 International license.

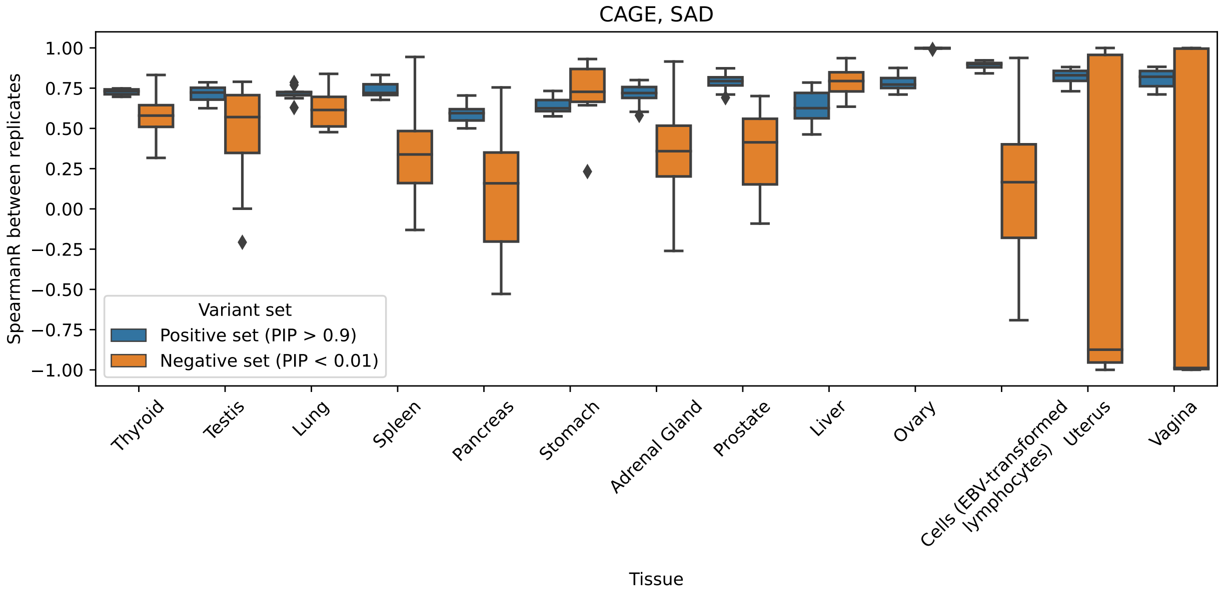

Figure S4: **Replicates are more consistent in predicting the effects of fine-mapped eQTLs than the effects of variants in a matched negative set.** For both sets of variants in each tissue, we calculate the Spearman correlation between predicted SAD (SNP Activity Difference) scores for every pair of replicates and show the distribution of correlations.

bioRxiv preprint doi: https://doi.org/10.1101/2023.12.21.572730; this version posted December 23, 2023. The copyright holder for this preprint (which was not certified by peer review) is the author/funder, who has granted bioRxiv a license to display the preprint in perpetuity. It is made available under aCC-BY 4.0 International license.

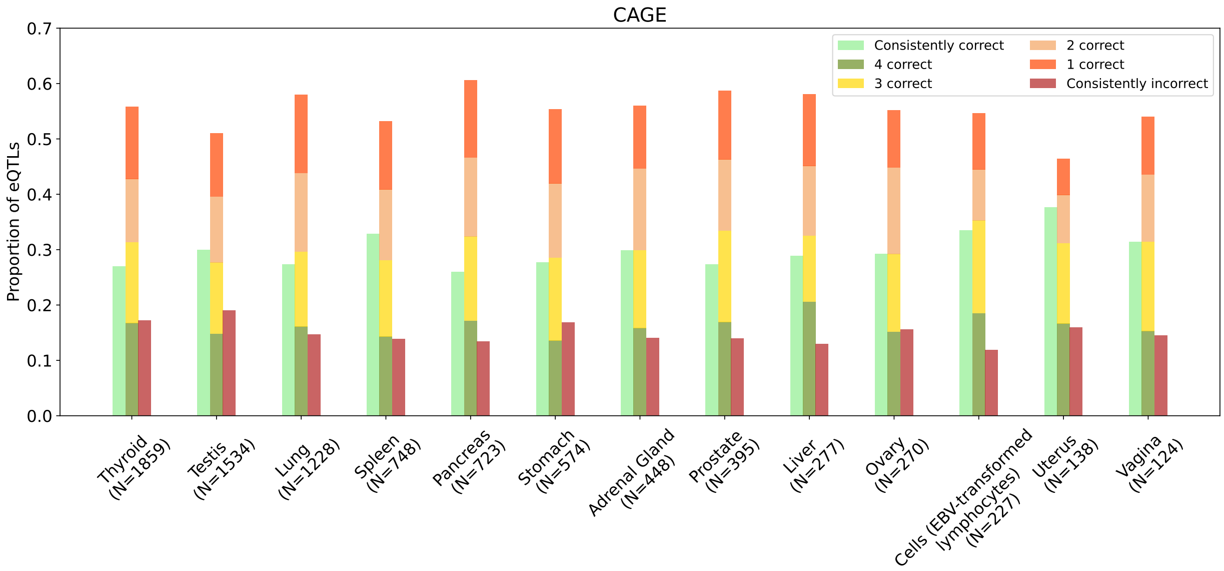

Figure S5: **In all tissues, replicates have have inconsistent sign predictions for 50-60% of fine-mapped eQTLs when using the tissue-matched CAGE track**. We stratify the plot in Fig. 3a by tissue and find similar proportions of consistently correct, inconsistent, and consistently incorrect sign predictions in all tissues.

bioRxiv preprint doi: https://doi.org/10.1101/2023.12.21.572730; this version posted December 23, 2023. The copyright holder for this preprint (which was not certified by peer review) is the author/funder, who has granted bioRxiv a license to display the preprint in perpetuity. It is made available under aCC-BY 4.0 International license.

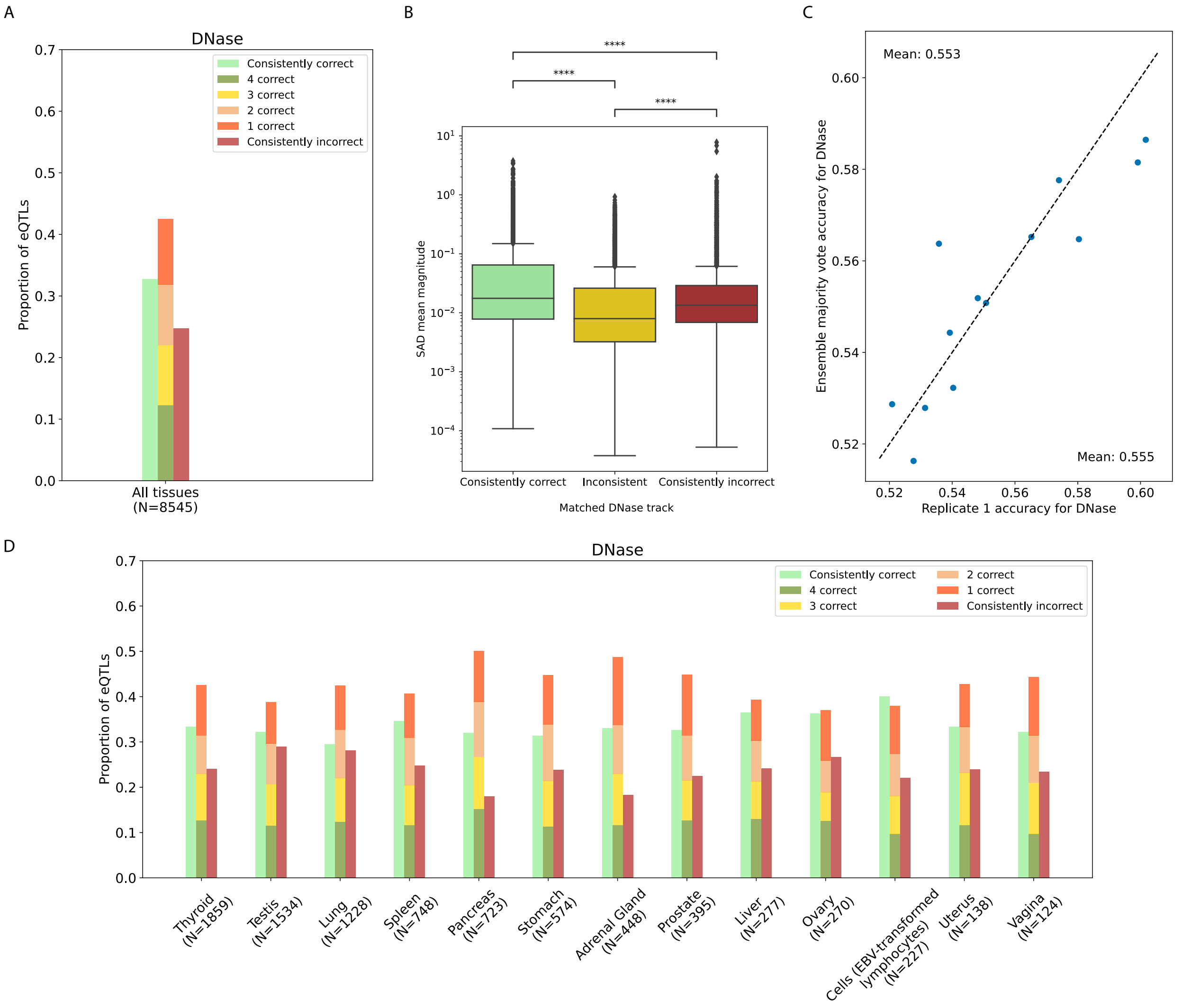

Figure S6: **eQTL sign prediction for DNase has some inconsistency across replicates, but less than for CAGE.** (a) We show the proportion of fine-mapped eQTLs with consistently correct, inconsistent, and consistently incorrect replicate predictions for eQTL effect sign. Pooled across tissues, about 32% of eQTLs are predicted consistently correctly, while about 25% are predicted consistently incorrectly. About 43% are inconsistently predicted. Of the consistently predicted eQTLs, about 65% are consistently correct, similar to the finding for CAGE. (b) eQTLs with inconsistent sign predictions across replicates tend to have smaller predicted effect sizes (mean of SAD score magnitude across replicates), for the tissue-matched DNase track. (c) A comparison of accuracy for eQTL sign prediction shows that the ensemble majority vote does not outperform a single replicate. Each point is the fine-mapped eQTL set of a different tissue. (d) We show the breakdown across tissues using matched DNase tracks. Across all tissues, about 40-50% of eQTLs have inconsistently predicted sign. About 30-40% eQTLs are predicted consistently correctly.

bioRxiv preprint doi: https://doi.org/10.1101/2023.12.21.572730; this version posted December 23, 2023. The copyright holder for this preprint (which was not certified by peer review) is the author/funder, who has granted bioRxiv a license to display the preprint in perpetuity. It is made available under aCC-BY 4.0 International license.

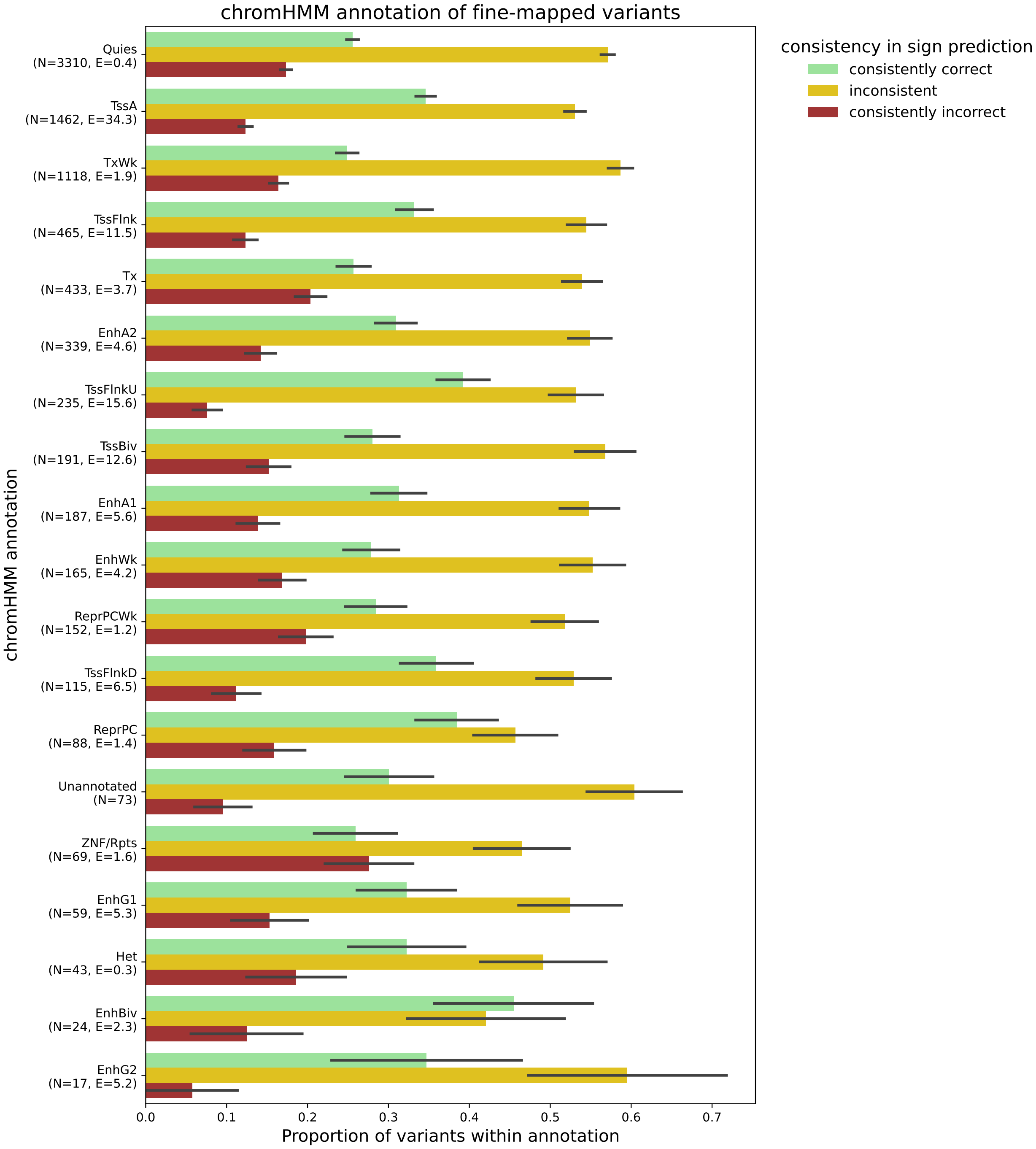

Figure S7: **Replicate consistency in sign prediction of fine-mapped eQTLs segmented by chromHMM annotation.** We labeled each fine-mapped variant with its tissue-matched chromHMM annotation or as "Unannotated" if it did not overlap a chromHMM annotation. For each annotation, we show the proportion of variants whose signs are predicted consistently correctly, inconsistently, or consistently incorrectly using the tissue-matched CAGE track. For all annotations, at least 40% of variants have inconsistent sign predictions. N denotes the number of fine-mapped variants overlapping that annotation, and E indicates the mean enrichment of fine-mapped variants in that annotation, averaged over the 13 examined tissues. Annotations are sorted by N. One standard deviation error bars are computed using 1000 bootstraps.

bioRxiv preprint doi: https://doi.org/10.1101/2023.12.21.572730; this version posted December 23, 2023. The copyright holder for this preprint (which was not certified by peer review) is the author/funder, who has granted bioRxiv a license to display the preprint in perpetuity. It is made available under aCC-BY 4.0 International license.

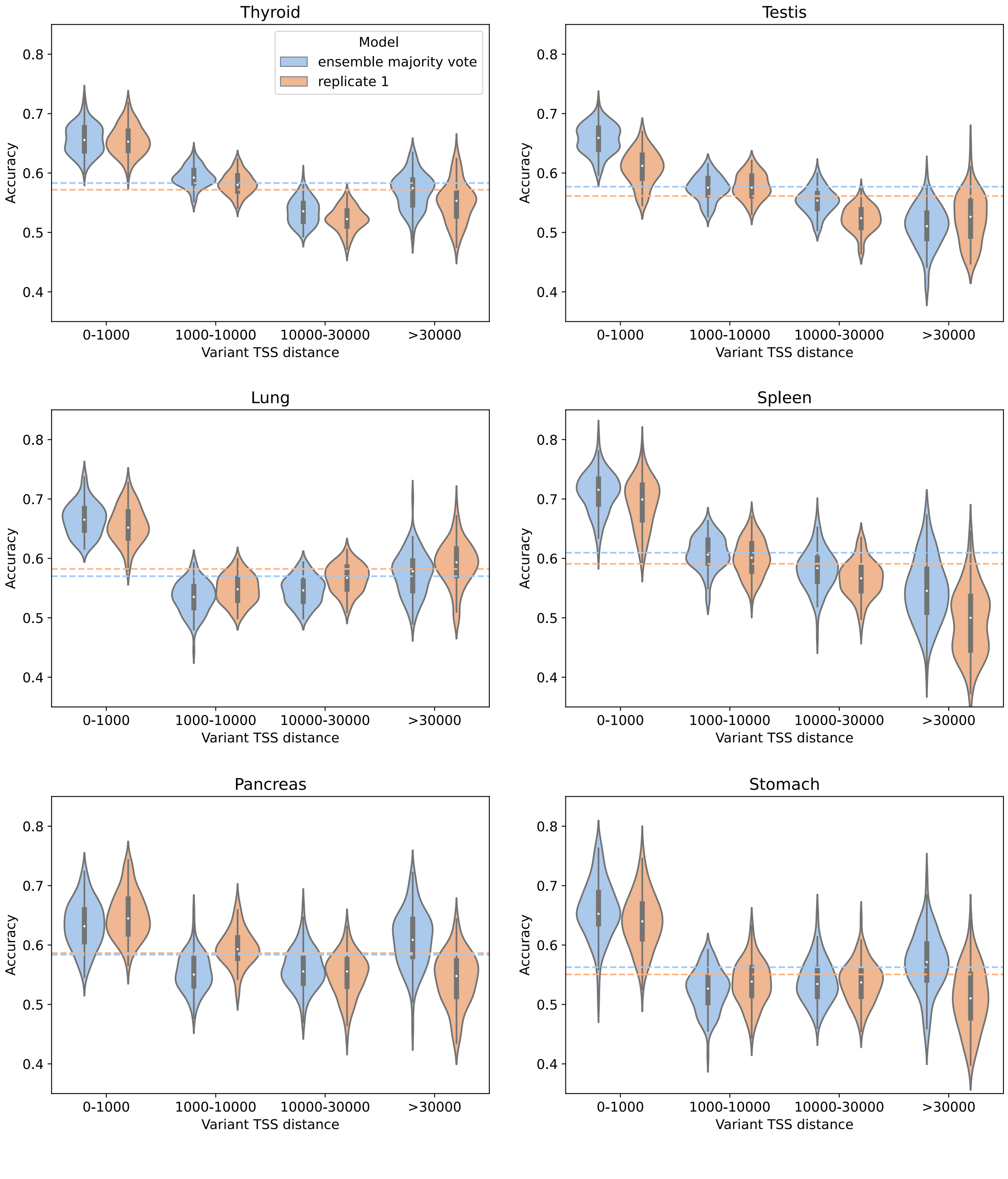

Figure S8: **Ensemble majority vote for CAGE predictions often performs better on the subset of proximal variants.** For the six tissues with the largest number of fine-mapped eQTLs, we stratify variants into TSS distance bins and compare eQTL sign prediction accuracy of a single replicate and the ensemble majority vote, using predictions from the tissue-matched CAGE track. Violinplots show the accuracy distributions over 100 bootstrap samples and dashed lines indicate the mean accuracies of the two models across all bins. In general, the ensemble majority vote has higher accuracy in the proximal TSS bin but not across all the bins. Across tissues, accuracy is highest for both models in the proximal TSS bin.
